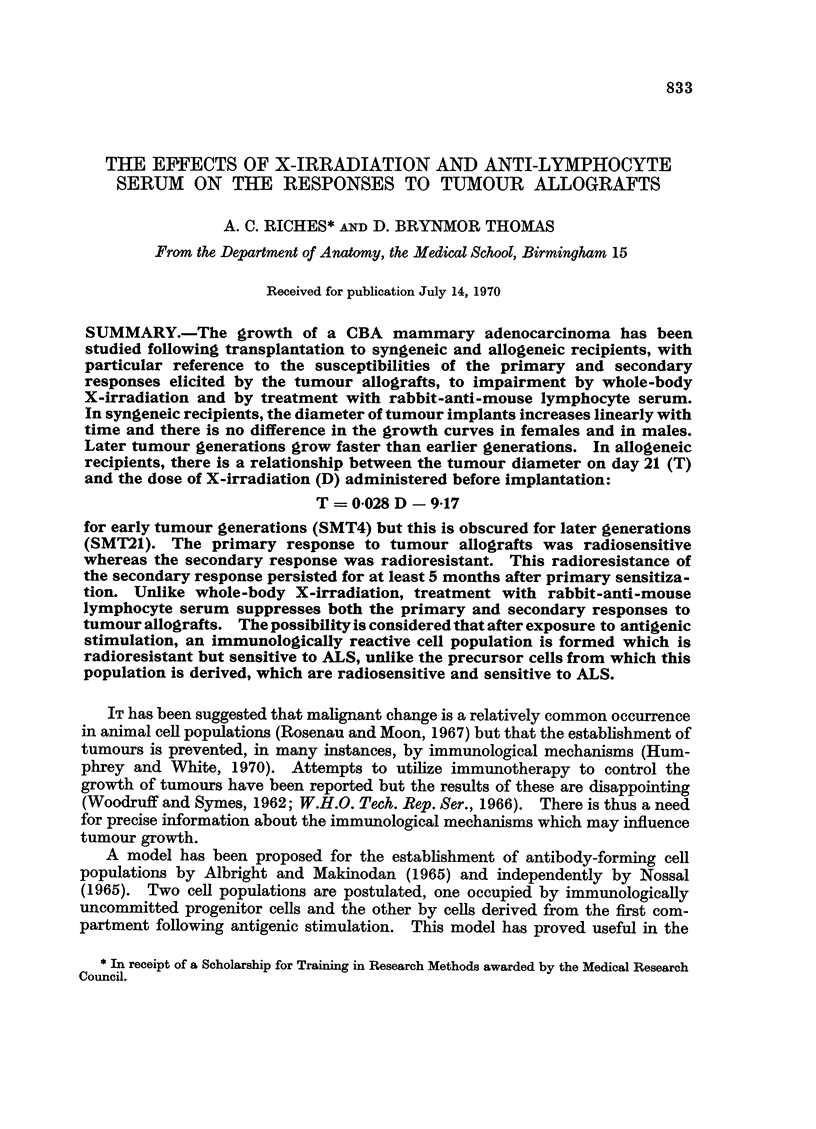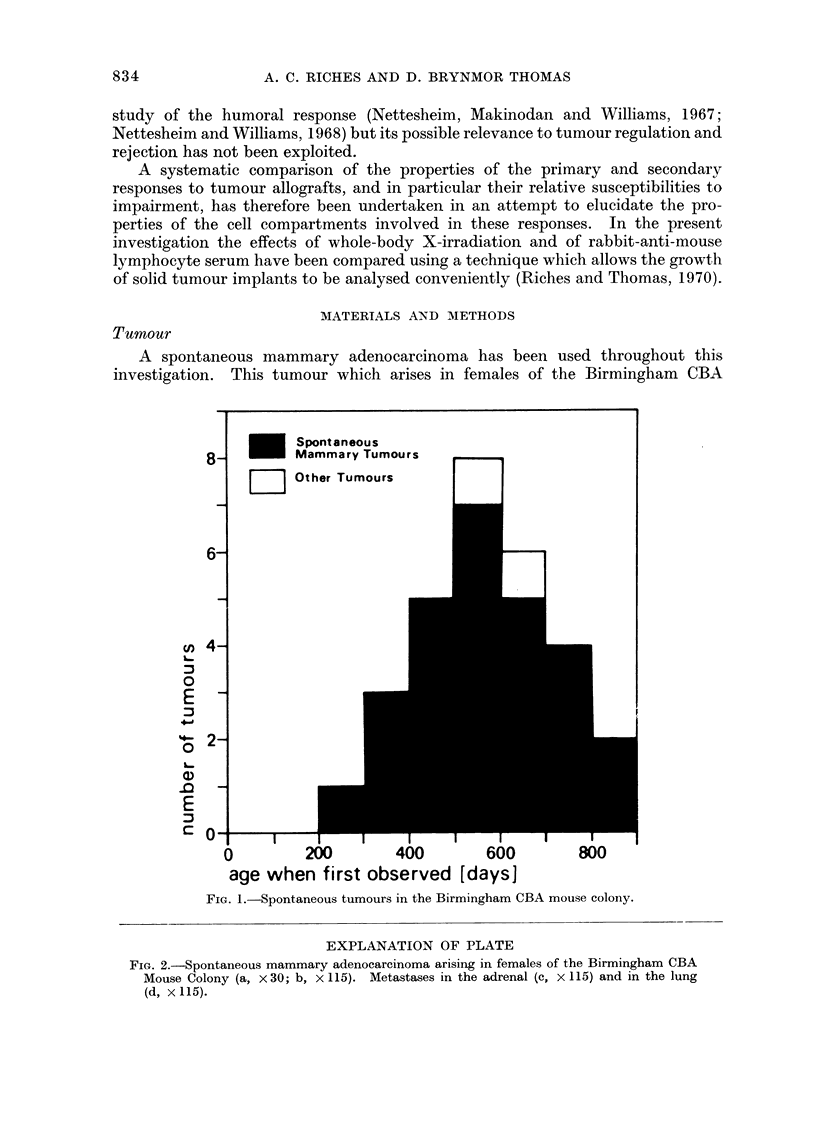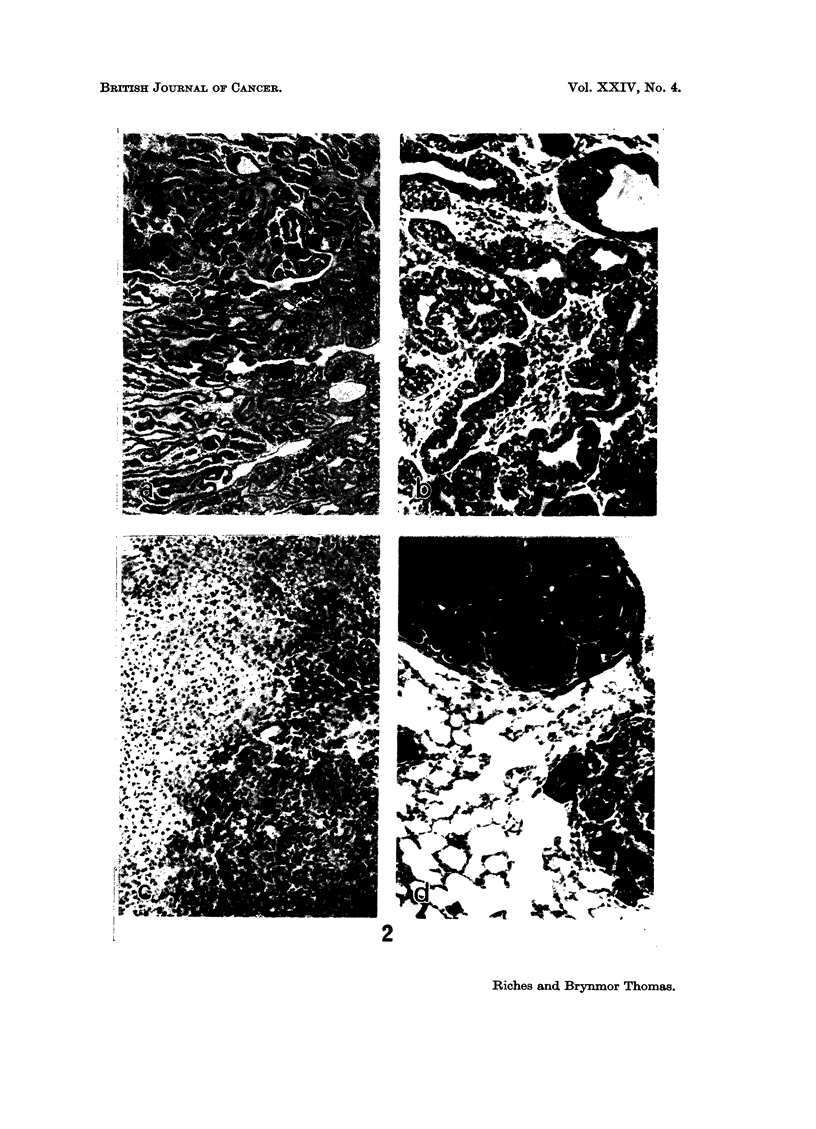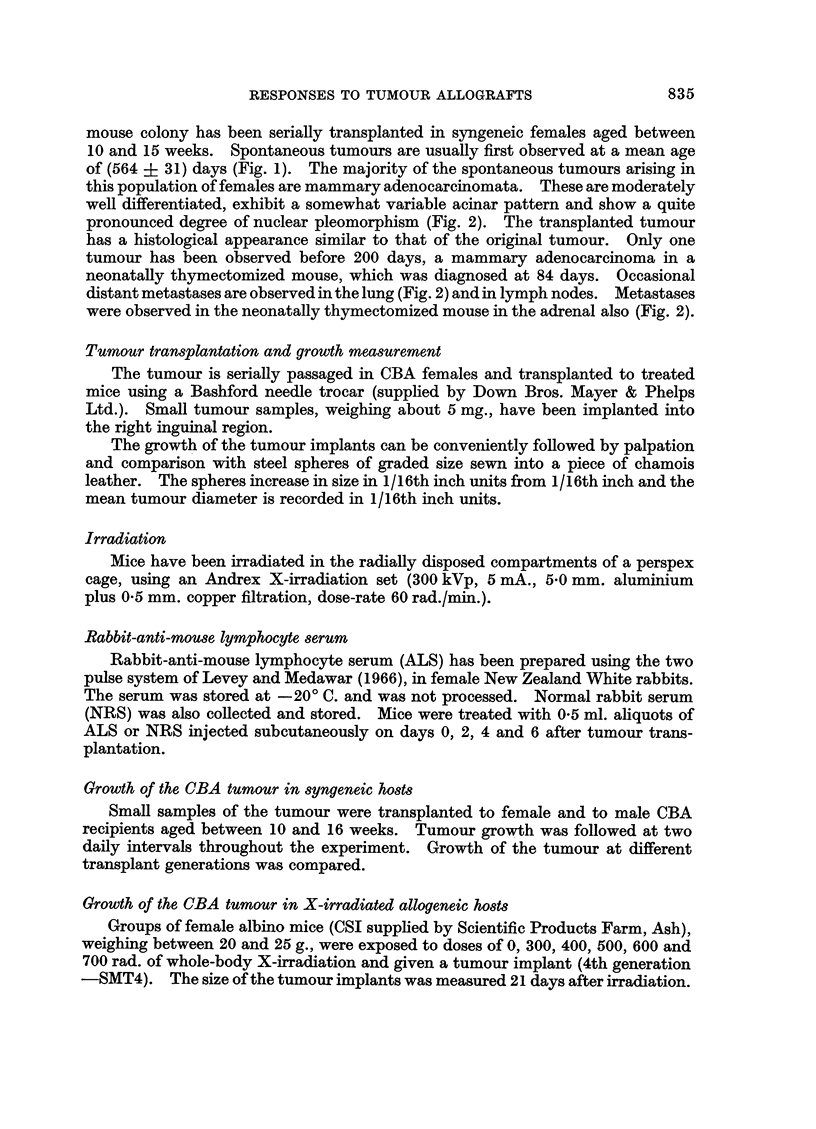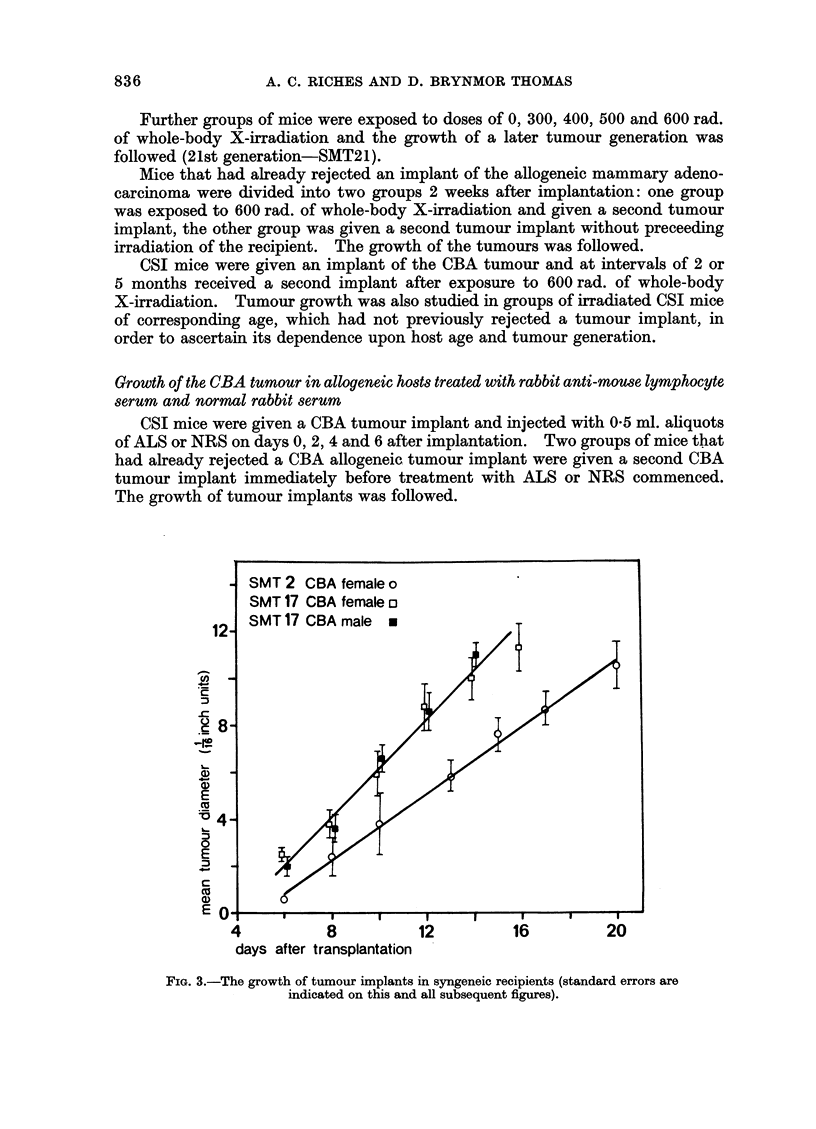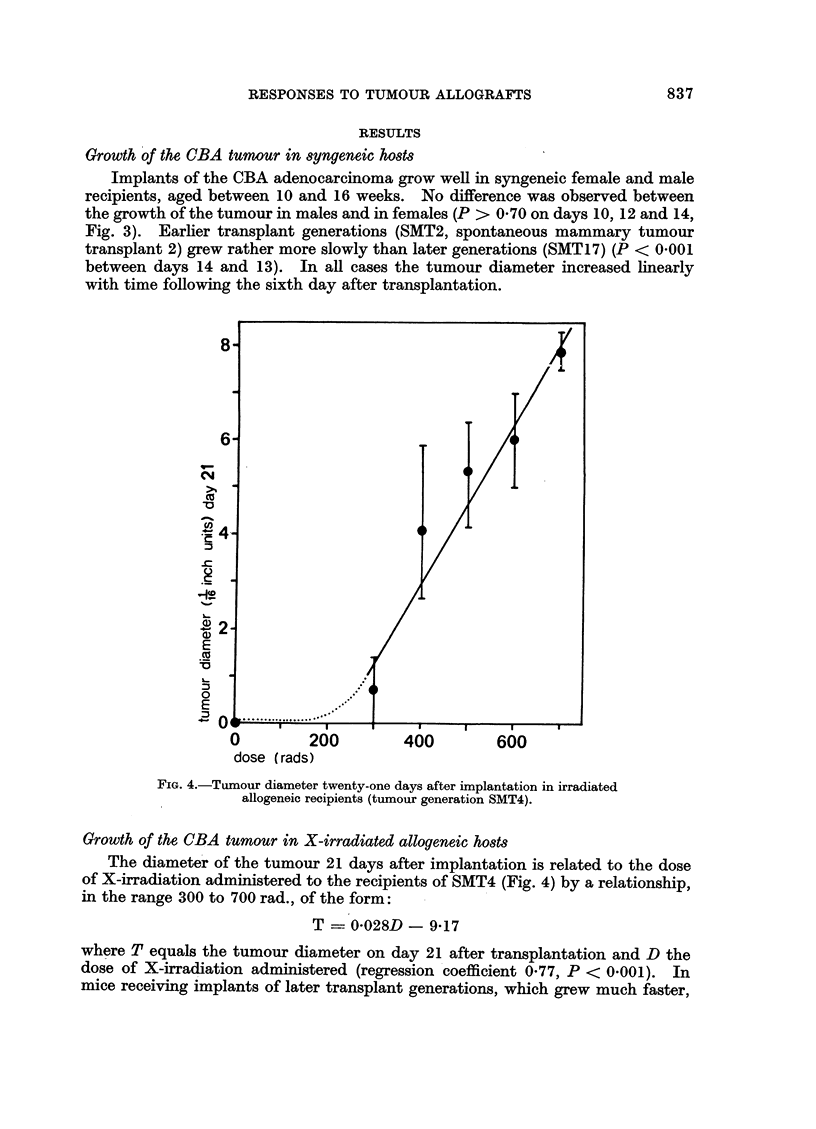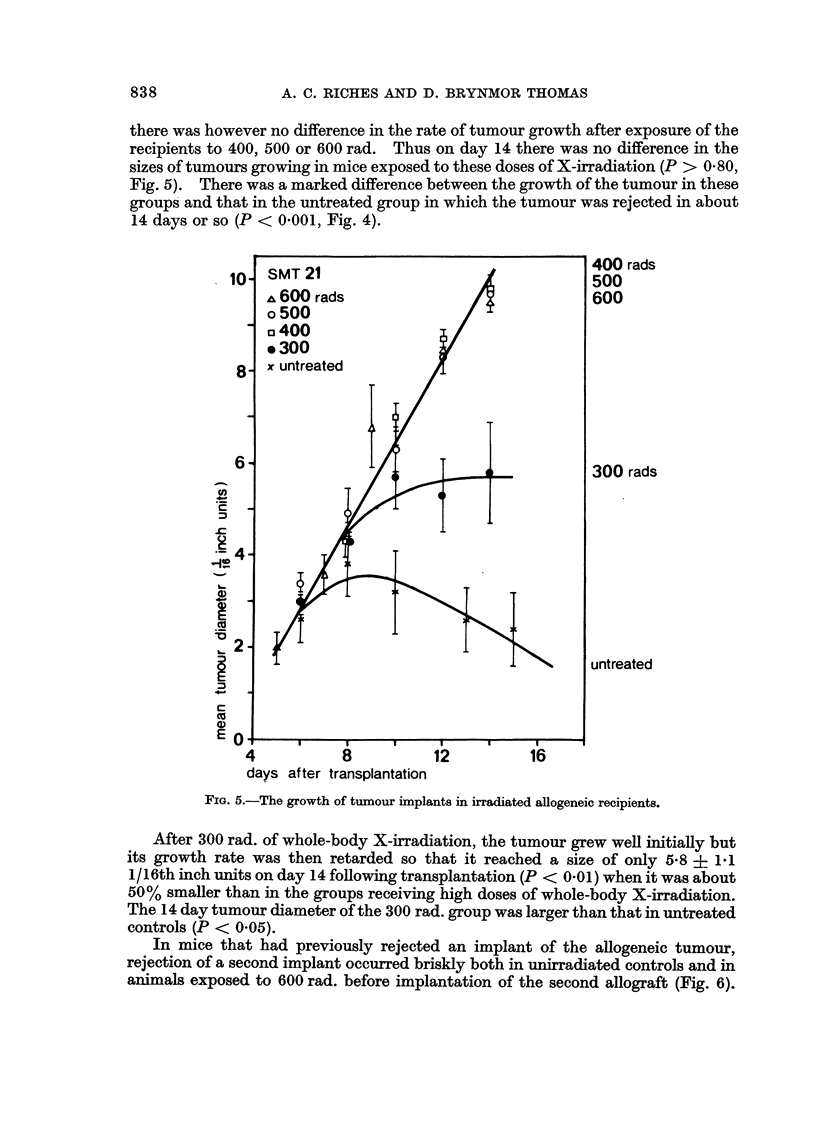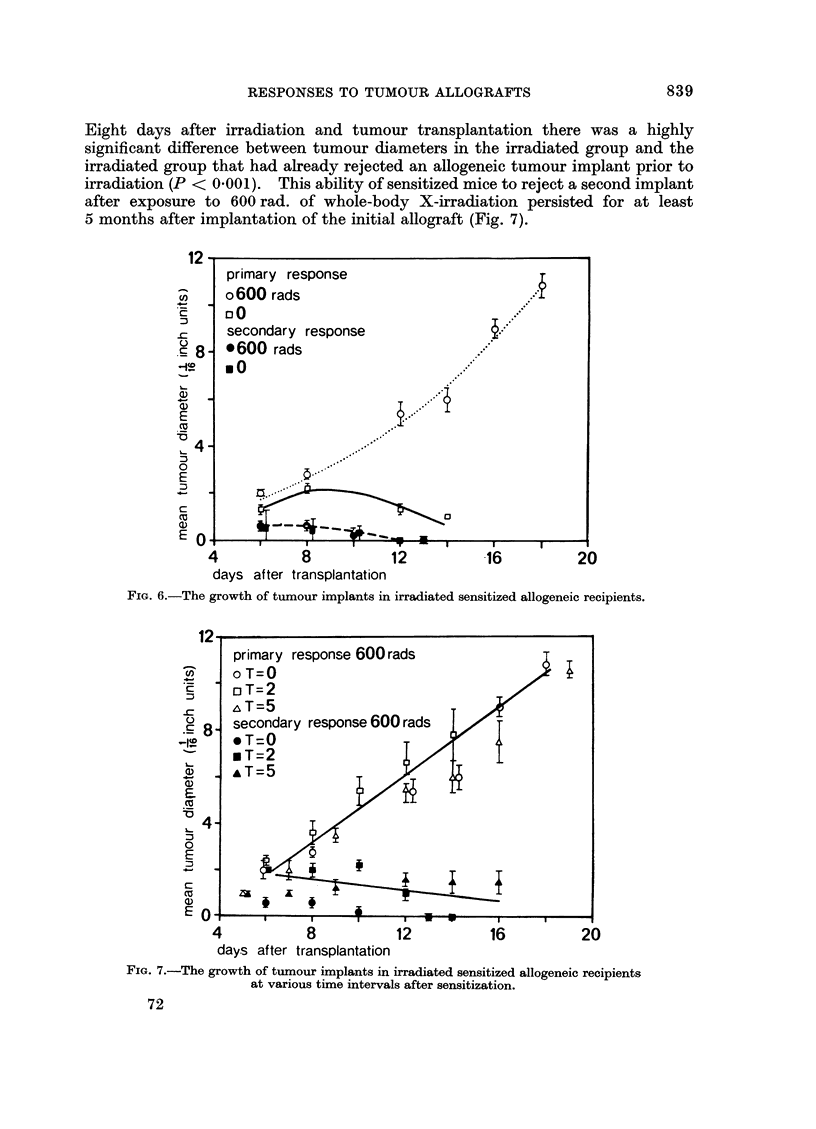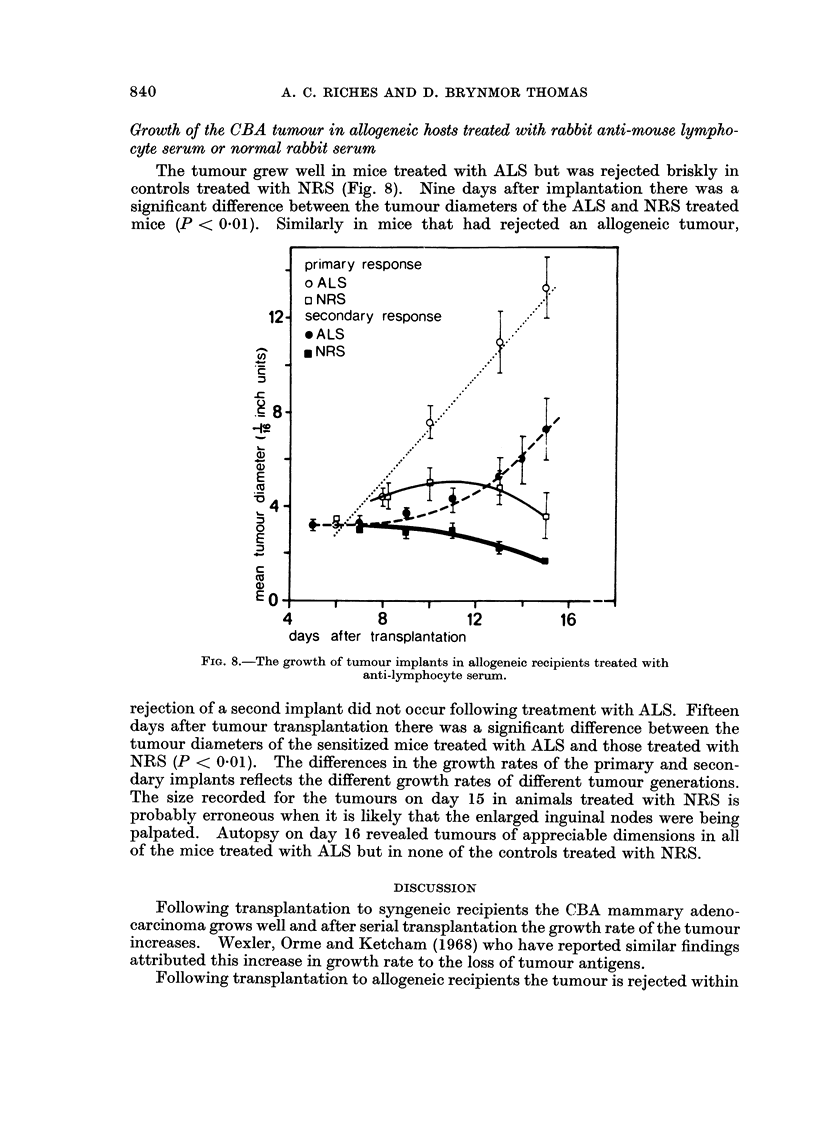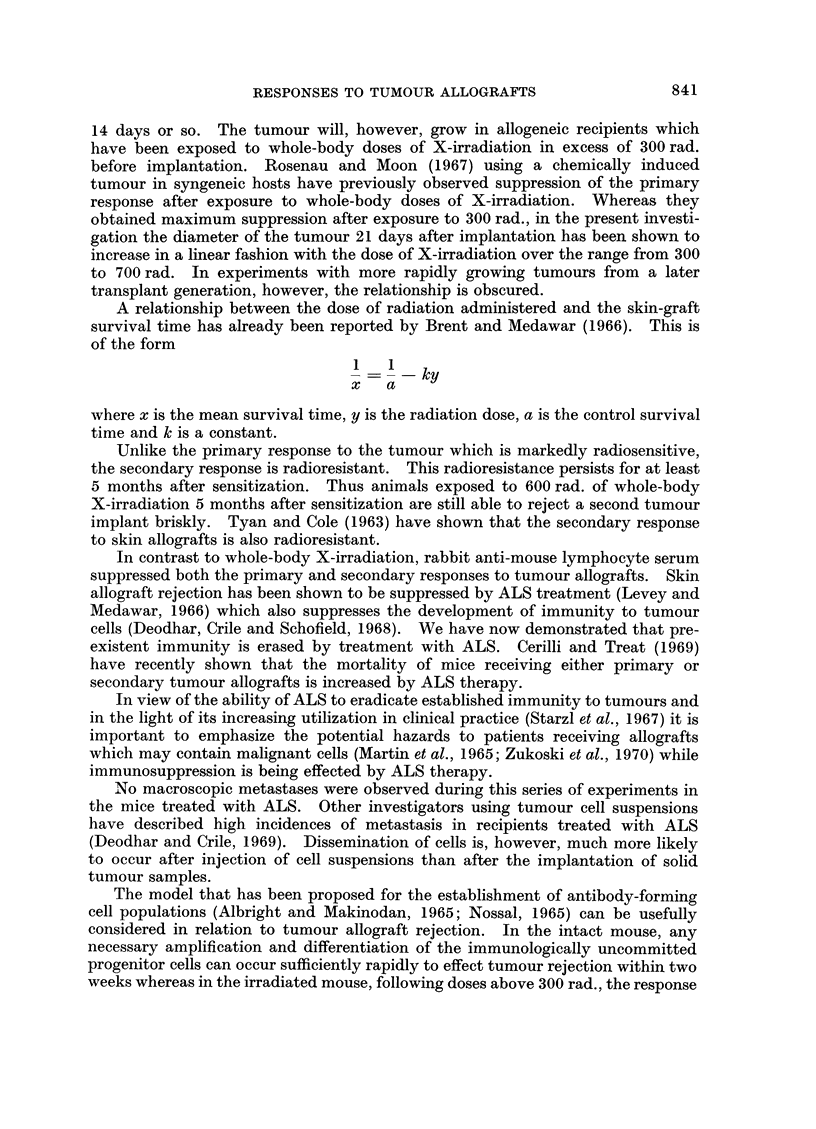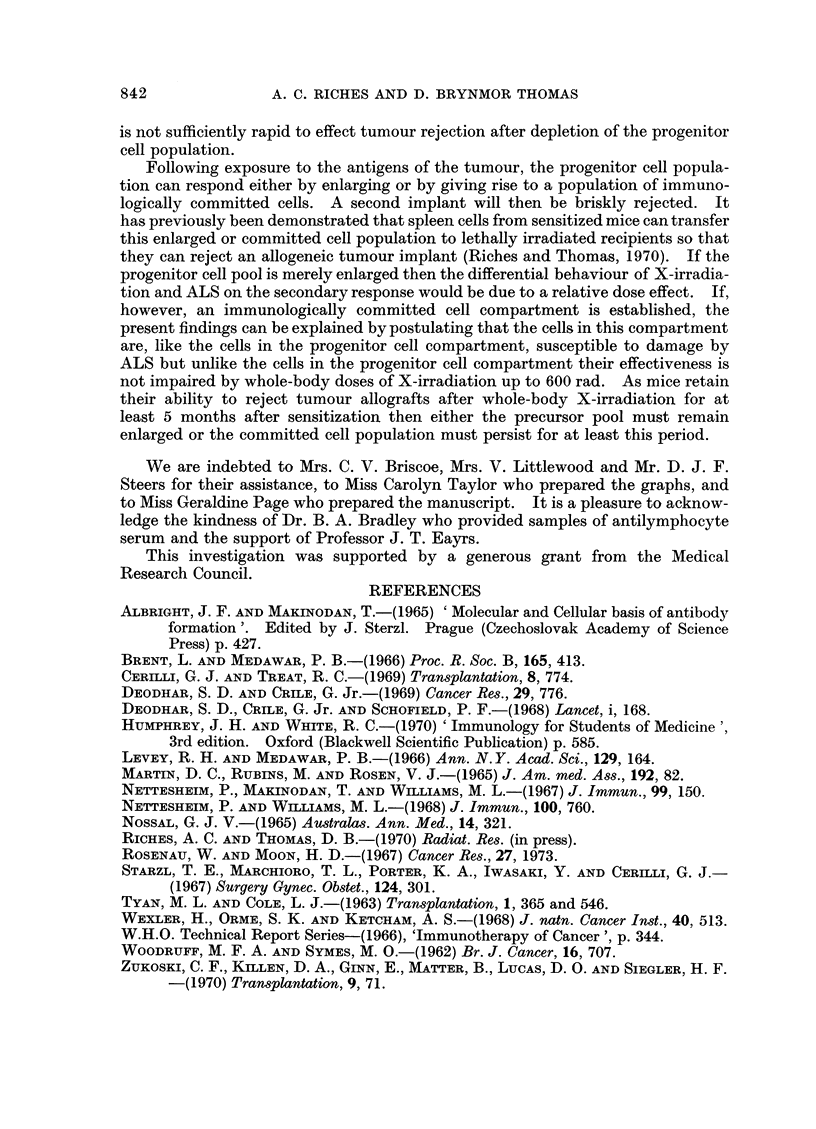# The Effects of X-irradiation and Anti-lymphocyte Serum on the Responses to Tumour Allografts

**DOI:** 10.1038/bjc.1970.98

**Published:** 1970-12

**Authors:** A. C. Riches, D. Brynmor Thomas

## Abstract

**Images:**


### THE EFFECTS OF X-IRRADIATION AND ANTI-LYMPHOCYTE SERUM ON THE RESPONSES TO TUMOUR ALLOGRAFTS

A. C. RICHES* AND D. BRYNMOR THOMAS

*From the Department of Anatomy, the Medical School, Birmingham* 15

Received for publication July 14, 1970

**SUMMARY.—The growth of a CBA mammary adenocarcinoma has been studied following transplantation to syngeneic and allogeneic recipients, with particular reference to the susceptibilities of the primary and secondary responses elicited by the tumour allografts, to impairment by whole-body X-irradiation and by treatment with rabbit-anti-mouse lymphocyte serum. In syngeneic recipients, the diameter of tumour implants increases linearly with time and there is no difference in the growth curves in females and in males. Later tumour generations grow faster than earlier generations. In allogeneic recipients, there is a relationship between the tumour diameter on day 21 (T) and the dose of X-irradiation (D) administered before implantation:**

$$T = 0{\cdot}028\ D - 9{\cdot}17$$

**for early tumour generations (SMT4) but this is obscured for later generations (SMT21). The primary response to tumour allografts was radiosensitive whereas the secondary response was radioresistant. This radioresistance of the secondary response persisted for at least 5 months after primary sensitization. Unlike whole-body X-irradiation, treatment with rabbit-anti-mouse lymphocyte serum suppresses both the primary and secondary responses to tumour allografts. The possibility is considered that after exposure to antigenic stimulation, an immunologically reactive cell population is formed which is radioresistant but sensitive to ALS, unlike the precursor cells from which this population is derived, which are radiosensitive and sensitive to ALS.**

It has been suggested that malignant change is a relatively common occurrence in animal cell populations (Rosenau and Moon, 1967) but that the establishment of tumours is prevented, in many instances, by immunological mechanisms (Humphrey and White, 1970). Attempts to utilize immunotherapy to control the growth of tumours have been reported but the results of these are disappointing (Woodruff and Symes, 1962; *W.H.O. Tech. Rep. Ser.*, 1966). There is thus a need for precise information about the immunological mechanisms which may influence tumour growth.

A model has been proposed for the establishment of antibody-forming cell populations by Albright and Makinodan (1965) and independently by Nossal (1965). Two cell populations are postulated, one occupied by immunologically uncommitted progenitor cells and the other by cells derived from the first compartment following antigenic stimulation. This model has proved useful in the

* In receipt of a Scholarship for Training in Research Methods awarded by the Medical Research Council.

study of the humoral response (Nettesheim, Makinodan and Williams, 1967; Nettesheim and Williams, 1968) but its possible relevance to tumour regulation and rejection has not been exploited.

A systematic comparison of the properties of the primary and secondary responses to tumour allografts, and in particular their relative susceptibilities to impairment, has therefore been undertaken in an attempt to elucidate the properties of the cell compartments involved in these responses. In the present investigation the effects of whole-body X-irradiation and of rabbit-anti-mouse lymphocyte serum have been compared using a technique which allows the growth of solid tumour implants to be analysed conveniently (Riches and Thomas, 1970).

### MATERIALS AND METHODS

### *Tumour*

A spontaneous mammary adenocarcinoma has been used throughout this investigation. This tumour which arises in females of the Birmingham CBA

FIG. 1.—Spontaneous tumours in the Birmingham CBA mouse colony.

### EXPLANATION OF PLATE

FIG. 2.—Spontaneous mammary adenocarcinoma arising in females of the Birmingham CBA Mouse Colony (a, ×30; b, ×115). Metastases in the adrenal (c, ×115) and in the lung (d, ×115).

2

Riches and Brynmor Thomas.

mouse colony has been serially transplanted in syngeneic females aged between 10 and 15 weeks. Spontaneous tumours are usually first observed at a mean age of (564 $\pm$ 31) days (Fig. 1). The majority of the spontaneous tumours arising in this population of females are mammary adenocarcinomata. These are moderately well differentiated, exhibit a somewhat variable acinar pattern and show a quite pronounced degree of nuclear pleomorphism (Fig. 2). The transplanted tumour has a histological appearance similar to that of the original tumour. Only one tumour has been observed before 200 days, a mammary adenocarcinoma in a neonatally thymectomized mouse, which was diagnosed at 84 days. Occasional distant metastases are observed in the lung (Fig. 2) and in lymph nodes. Metastases were observed in the neonatally thymectomized mouse in the adrenal also (Fig. 2).

*Tumour transplantation and growth measurement*

The tumour is serially passaged in CBA females and transplanted to treated mice using a Bashford needle trocar (supplied by Down Bros. Mayer & Phelps Ltd.). Small tumour samples, weighing about 5 mg., have been implanted into the right inguinal region.

The growth of the tumour implants can be conveniently followed by palpation and comparison with steel spheres of graded size sewn into a piece of chamois leather. The spheres increase in size in 1/16th inch units from 1/16th inch and the mean tumour diameter is recorded in 1/16th inch units.

*Irradiation*

Mice have been irradiated in the radially disposed compartments of a perspex cage, using an Andrex X-irradiation set (300 kVp, 5 mA., 5·0 mm. aluminium plus 0·5 mm. copper filtration, dose-rate 60 rad./min.).

*Rabbit-anti-mouse lymphocyte serum*

Rabbit-anti-mouse lymphocyte serum (ALS) has been prepared using the two pulse system of Levey and Medawar (1966), in female New Zealand White rabbits. The serum was stored at $-20°$ C. and was not processed. Normal rabbit serum (NRS) was also collected and stored. Mice were treated with 0·5 ml. aliquots of ALS or NRS injected subcutaneously on days 0, 2, 4 and 6 after tumour transplantation.

*Growth of the CBA tumour in syngeneic hosts*

Small samples of the tumour were transplanted to female and to male CBA recipients aged between 10 and 16 weeks. Tumour growth was followed at two daily intervals throughout the experiment. Growth of the tumour at different transplant generations was compared.

*Growth of the CBA tumour in X-irradiated allogeneic hosts*

Groups of female albino mice (CSI supplied by Scientific Products Farm, Ash), weighing between 20 and 25 g., were exposed to doses of 0, 300, 400, 500, 600 and 700 rad. of whole-body X-irradiation and given a tumour implant (4th generation —SMT4). The size of the tumour implants was measured 21 days after irradiation.

Further groups of mice were exposed to doses of 0, 300, 400, 500 and 600 rad. of whole-body X-irradiation and the growth of a later tumour generation was followed (21st generation—SMT21).

Mice that had already rejected an implant of the allogeneic mammary adenocarcinoma were divided into two groups 2 weeks after implantation: one group was exposed to 600 rad. of whole-body X-irradiation and given a second tumour implant, the other group was given a second tumour implant without preceeding irradiation of the recipient. The growth of the tumours was followed.

CSI mice were given an implant of the CBA tumour and at intervals of 2 or 5 months received a second implant after exposure to 600 rad. of whole-body X-irradiation. Tumour growth was also studied in groups of irradiated CSI mice of corresponding age, which had not previously rejected a tumour implant, in order to ascertain its dependence upon host age and tumour generation.

*Growth of the CBA tumour in allogeneic hosts treated with rabbit anti-mouse lymphocyte serum and normal rabbit serum*

CSI mice were given a CBA tumour implant and injected with 0·5 ml. aliquots of ALS or NRS on days 0, 2, 4 and 6 after implantation. Two groups of mice that had already rejected a CBA allogeneic tumour implant were given a second CBA tumour implant immediately before treatment with ALS or NRS commenced. The growth of tumour implants was followed.

FIG. 3.—The growth of tumour implants in syngeneic recipients (standard errors are indicated on this and all subsequent figures).

### RESULTS

### *Growth of the CBA tumour in syngeneic hosts*

Implants of the CBA adenocarcinoma grow well in syngeneic female and male recipients, aged between 10 and 16 weeks. No difference was observed between the growth of the tumour in males and in females ($P > 0{\cdot}70$ on days 10, 12 and 14, Fig. 3). Earlier transplant generations (SMT2, spontaneous mammary tumour transplant 2) grew rather more slowly than later generations (SMT17) ($P < 0{\cdot}001$ between days 14 and 13). In all cases the tumour diameter increased linearly with time following the sixth day after transplantation.

Fig. 4.—Tumour diameter twenty-one days after implantation in irradiated allogeneic recipients (tumour generation SMT4).

### *Growth of the CBA tumour in X-irradiated allogeneic hosts*

The diameter of the tumour 21 days after implantation is related to the dose of X-irradiation administered to the recipients of SMT4 (Fig. 4) by a relationship, in the range 300 to 700 rad., of the form:

$$T = 0{\cdot}028D - 9{\cdot}17$$

where $T$ equals the tumour diameter on day 21 after transplantation and $D$ the dose of X-irradiation administered (regression coefficient $0{\cdot}77$, $P < 0{\cdot}001$). In mice receiving implants of later transplant generations, which grew much faster, there was however no difference in the rate of tumour growth after exposure of the recipients to 400, 500 or 600 rad. Thus on day 14 there was no difference in the sizes of tumours growing in mice exposed to these doses of X-irradiation ($P > 0{\cdot}80$, Fig. 5). There was a marked difference between the growth of the tumour in these groups and that in the untreated group in which the tumour was rejected in about 14 days or so ($P < 0{\cdot}001$, Fig. 4).

FIG. 5.—The growth of tumour implants in irradiated allogeneic recipients.

After 300 rad. of whole-body X-irradiation, the tumour grew well initially but its growth rate was then retarded so that it reached a size of only $5{\cdot}8 \pm 1{\cdot}1$ 1/16th inch units on day 14 following transplantation ($P < 0{\cdot}01$) when it was about 50% smaller than in the groups receiving high doses of whole-body X-irradiation. The 14 day tumour diameter of the 300 rad. group was larger than that in untreated controls ($P < 0{\cdot}05$).

In mice that had previously rejected an implant of the allogeneic tumour, rejection of a second implant occurred briskly both in unirradiated controls and in animals exposed to 600 rad. before implantation of the second allograft (Fig. 6).

Eight days after irradiation and tumour transplantation there was a highly significant difference between tumour diameters in the irradiated group and the irradiated group that had already rejected an allogeneic tumour implant prior to irradiation ($P < 0{\cdot}001$). This ability of sensitized mice to reject a second implant after exposure to 600 rad. of whole-body X-irradiation persisted for at least 5 months after implantation of the initial allograft (Fig. 7).

Fig. 6.—The growth of tumour implants in irradiated sensitized allogeneic recipients.

Fig. 7.—The growth of tumour implants in irradiated sensitized allogeneic recipients at various time intervals after sensitization.

*Growth of the CBA tumour in allogeneic hosts treated with rabbit anti-mouse lymphocyte serum or normal rabbit serum*

The tumour grew well in mice treated with ALS but was rejected briskly in controls treated with NRS (Fig. 8). Nine days after implantation there was a significant difference between the tumour diameters of the ALS and NRS treated mice ($P < 0{\cdot}01$). Similarly in mice that had rejected an allogeneic tumour,

FIG. 8.—The growth of tumour implants in allogeneic recipients treated with anti-lymphocyte serum.

rejection of a second implant did not occur following treatment with ALS. Fifteen days after tumour transplantation there was a significant difference between the tumour diameters of the sensitized mice treated with ALS and those treated with NRS ($P < 0{\cdot}01$). The differences in the growth rates of the primary and secondary implants reflects the different growth rates of different tumour generations. The size recorded for the tumours on day 15 in animals treated with NRS is probably erroneous when it is likely that the enlarged inguinal nodes were being palpated. Autopsy on day 16 revealed tumours of appreciable dimensions in all of the mice treated with ALS but in none of the controls treated with NRS.

### DISCUSSION

Following transplantation to syngeneic recipients the CBA mammary adenocarcinoma grows well and after serial transplantation the growth rate of the tumour increases. Wexler, Orme and Ketcham (1968) who have reported similar findings attributed this increase in growth rate to the loss of tumour antigens.

Following transplantation to allogeneic recipients the tumour is rejected within

14 days or so. The tumour will, however, grow in allogeneic recipients which have been exposed to whole-body doses of X-irradiation in excess of 300 rad. before implantation. Rosenau and Moon (1967) using a chemically induced tumour in syngeneic hosts have previously observed suppression of the primary response after exposure to whole-body doses of X-irradiation. Whereas they obtained maximum suppression after exposure to 300 rad., in the present investigation the diameter of the tumour 21 days after implantation has been shown to increase in a linear fashion with the dose of X-irradiation over the range from 300 to 700 rad. In experiments with more rapidly growing tumours from a later transplant generation, however, the relationship is obscured.

A relationship between the dose of radiation administered and the skin-graft survival time has already been reported by Brent and Medawar (1966). This is of the form

$$\frac{1}{x} = \frac{1}{a} - ky$$

where $x$ is the mean survival time, $y$ is the radiation dose, $a$ is the control survival time and $k$ is a constant.

Unlike the primary response to the tumour which is markedly radiosensitive, the secondary response is radioresistant. This radioresistance persists for at least 5 months after sensitization. Thus animals exposed to 600 rad. of whole-body X-irradiation 5 months after sensitization are still able to reject a second tumour implant briskly. Tyan and Cole (1963) have shown that the secondary response to skin allografts is also radioresistant.

In contrast to whole-body X-irradiation, rabbit anti-mouse lymphocyte serum suppressed both the primary and secondary responses to tumour allografts. Skin allograft rejection has been shown to be suppressed by ALS treatment (Levey and Medawar, 1966) which also suppresses the development of immunity to tumour cells (Deodhar, Crile and Schofield, 1968). We have now demonstrated that pre-existent immunity is erased by treatment with ALS. Cerilli and Treat (1969) have recently shown that the mortality of mice receiving either primary or secondary tumour allografts is increased by ALS therapy.

In view of the ability of ALS to eradicate established immunity to tumours and in the light of its increasing utilization in clinical practice (Starzl *et al.*, 1967) it is important to emphasize the potential hazards to patients receiving allografts which may contain malignant cells (Martin *et al.*, 1965; Zukoski *et al.*, 1970) while immunosuppression is being effected by ALS therapy.

No macroscopic metastases were observed during this series of experiments in the mice treated with ALS. Other investigators using tumour cell suspensions have described high incidences of metastasis in recipients treated with ALS (Deodhar and Crile, 1969). Dissemination of cells is, however, much more likely to occur after injection of cell suspensions than after the implantation of solid tumour samples.

The model that has been proposed for the establishment of antibody-forming cell populations (Albright and Makinodan, 1965; Nossal, 1965) can be usefully considered in relation to tumour allograft rejection. In the intact mouse, any necessary amplification and differentiation of the immunologically uncommitted progenitor cells can occur sufficiently rapidly to effect tumour rejection within two weeks whereas in the irradiated mouse, following doses above 300 rad., the response is not sufficiently rapid to effect tumour rejection after depletion of the progenitor cell population.

Following exposure to the antigens of the tumour, the progenitor cell population can respond either by enlarging or by giving rise to a population of immunologically committed cells. A second implant will then be briskly rejected. It has previously been demonstrated that spleen cells from sensitized mice can transfer this enlarged or committed cell population to lethally irradiated recipients so that they can reject an allogeneic tumour implant (Riches and Thomas, 1970). If the progenitor cell pool is merely enlarged then the differential behaviour of X-irradiation and ALS on the secondary response would be due to a relative dose effect. If, however, an immunologically committed cell compartment is established, the present findings can be explained by postulating that the cells in this compartment are, like the cells in the progenitor cell compartment, susceptible to damage by ALS but unlike the cells in the progenitor cell compartment their effectiveness is not impaired by whole-body doses of X-irradiation up to 600 rad. As mice retain their ability to reject tumour allografts after whole-body X-irradiation for at least 5 months after sensitization then either the precursor pool must remain enlarged or the committed cell population must persist for at least this period.

We are indebted to Mrs. C. V. Briscoe, Mrs. V. Littlewood and Mr. D. J. F. Steers for their assistance, to Miss Carolyn Taylor who prepared the graphs, and to Miss Geraldine Page who prepared the manuscript. It is a pleasure to acknowledge the kindness of Dr. B. A. Bradley who provided samples of antilymphocyte serum and the support of Professor J. T. Eayrs.

This investigation was supported by a generous grant from the Medical Research Council.

### REFERENCES

ALBRIGHT, J. F. AND MAKINODAN, T.—(1965) ' Molecular and Cellular basis of antibody formation '. Edited by J. Sterzl. Prague (Czechoslovak Academy of Science Press) p. 427.

BRENT, L. AND MEDAWAR, P. B.—(1966) *Proc. R. Soc.* B, **165**, 413.

CERILLI, G. J. AND TREAT, R. C.—(1969) *Transplantation*, **8**, 774.

DEODHAR, S. D. AND CRILE, G. Jr.—(1969) *Cancer Res.*, **29**, 776.

DEODHAR, S. D., CRILE, G. Jr. AND SCHOFIELD, P. F.—(1968) *Lancet*, i, 168.

HUMPHREY, J. H. AND WHITE, R. C.—(1970) ' Immunology for Students of Medicine ', 3rd edition. Oxford (Blackwell Scientific Publication) p. 585.

LEVEY, R. H. AND MEDAWAR, P. B.—(1966) *Ann. N.Y. Acad. Sci.*, **129**, 164.

MARTIN, D. C., RUBINS, M. AND ROSEN, V. J.—(1965) *J. Am. med. Ass.*, **192**, 82.

NETTESHEIM, P., MAKINODAN, T. AND WILLIAMS, M. L.—(1967) *J. Immun.*, **99**, 150.

NETTESHEIM, P. AND WILLIAMS, M. L.—(1968) *J. Immun.*, **100**, 760.

NOSSAL, G. J. V.—(1965) *Australas. Ann. Med.*, **14**, 321.

RICHES, A. C. AND THOMAS, D. B.—(1970) *Radiat. Res.* (in press).

ROSENAU, W. AND MOON, H. D.—(1967) *Cancer Res.*, **27**, 1973.

STARZL, T. E., MARCHIORO, T. L., PORTER, K. A., IWASAKI, Y. AND CERILLI, G. J.—(1967) *Surgery Gynec. Obstet.*, **124**, 301.

TYAN, M. L. AND COLE, L. J.—(1963) *Transplantation*, **1**, 365 and 546.

WEXLER, H., ORME, S. K. AND KETCHAM, A. S.—(1968) *J. natn. Cancer Inst.*, **40**, 513.

W.H.O. Technical Report Series—(1966), 'Immunotherapy of Cancer ', p. 344.

WOODRUFF, M. F. A. AND SYMES, M. O.—(1962) *Br. J. Cancer*, **16**, 707.

ZUKOSKI, C. F., KILLEN, D. A., GINN, E., MATTER, B., LUCAS, D. O. AND SIEGLER, H. F. —(1970) *Transplantation*, **9**, 71.